# P-722. Troponin I Levels on Admission as a Predictor of Clinical Outcomes in Respiratory Viral Infection

**DOI:** 10.1093/ofid/ofae631.918

**Published:** 2025-01-29

**Authors:** Robin Sherchan, Rosalba Gomez Morones, Miguel A Covarrubias Gonzalez, Daniela Morales-Aseff, Claudia Aguayo-Millan, Alonso Benjamin Arellano Lopez, Felipe Salinas Martinez, Evan Goldin, David Beenhouwer, Maria C Rodriguez-Barradas

**Affiliations:** Michael E. DeBakey VA Medical Center / Baylor College of Medicine, Houston, Texas; Baylor Collage of Medicine, Houston, Texas; Michael E. DeBakey VA Medical Center / Baylor College of Medicine, Houston, Texas; Baylor College of Medicine, Michael E. DeBakey VA Medical Center, Houston, Texas; Baylor College of Medicine, Michael E. DeBakey VA Medical Center, Houston, Texas; Michael E. DeBakey VA Medical Center / Baylor College of Medicine, Houston, Texas; Michael E. DeBakey VA Medical Center / Baylor College of Medicine, Houston, Texas; West Los Angeles VA Medical Center, Los Angeles, California; VA Greater Los Angeles, Los Angeles, CA; Michael E. DeBakey VAMC and Baylor College of Medicine, Houston, Texas

## Abstract

**Background:**

Cardiac troponin I (Tn) is a biomarker for myocardial injury, often elevated in acute coronary syndrome (ACS), sepsis, and acute respiratory viral infection (ARI). The significance of elevated Tn in patients admitted with ARI is not clear. We evaluated readmission due to cardiovascular (CV) events (ACS, heart failure (HF), or stroke) and all-cause mortality in hospitalized ARI patients with elevated Tn on admission.

**Figure:**
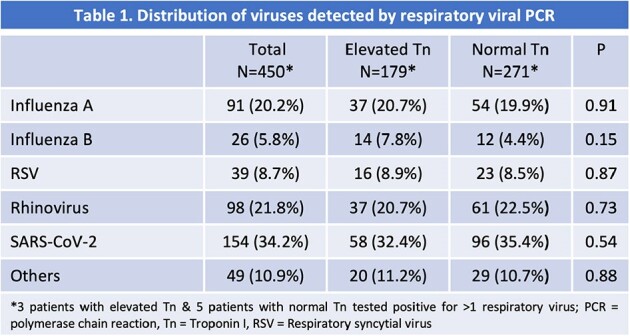

**Methods:**

This is a 2-VA site sub-study of SUPERNOVA (Surveillance Platform for Enteric and Respiratory Infectious Organisms in the VA), that included patients with positive nasopharyngeal viral polymerase chain reaction and Tn obtained within 48 hours of admission during 3 ARI seasons (S): S1, 10/2016-9/2017; S2, 10/2017-9/2018; and S3 11/2021-1/2022. Outcomes assessed were 30, 90, and 365-day all-cause mortality and 365-day readmissions due to CV events.

**Figure:**
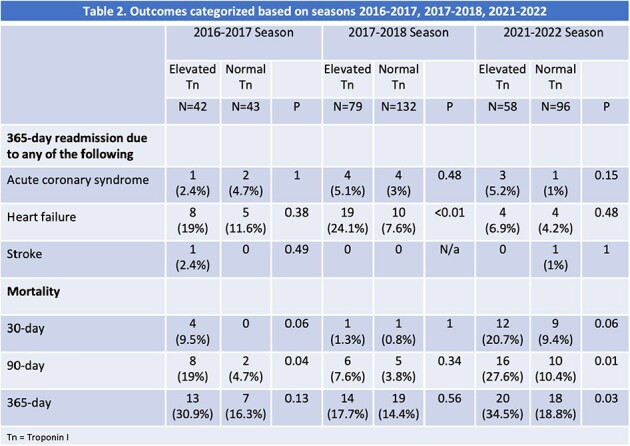

**Results:**

450 patients were included with >90% males. The mean age (years) and Charlson Comorbidity Index (CCI) for elevated Tn and normal Tn groups were 71 and 67, and 6.5 and 5.1, respectively. Rhinovirus (22%) and Influenza A (20%) were the predominant viruses in the first two seasons. All S3 patients had SARS-CoV-2 (34%) (Table 1). The elevated Tn group had significantly higher 90-day mortality in S1 and S3, and 365-day mortality in S3 (Table 2). For all seasons combined, the elevated Tn group had significantly higher 90-day mortality (17% vs 6%), 365-day mortality (26% vs 16%), and higher HF readmissions (18% vs 7%) than the normal Tn group (Table 3). When adjusted for CCI, the difference was not significant for 365-day mortality (p=0.29), but the composite outcome for 365-day mortality and readmission for HF (40% vs 22%) remained significant (p=0.02). The 365-day mortality was not significantly different for the elevated Tn group across seasons.

**Figure:**
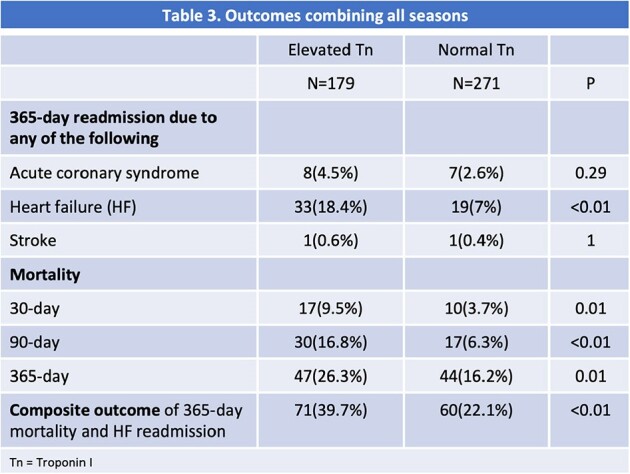

**Conclusion:**

ARI patients with elevated Tn had a higher risk of all-cause mortality and HF readmission within one year, which remained statistically significant after adjustment for comorbidities for the 90-day mortality and HF readmission. The composite outcome of 365-day mortality and HF readmission was significantly higher in the elevated Tn group. These findings underscore the prognostic value of Tn in predicting CV and mortality outcomes in hospitalized patients with ARI.

**Disclosures:**

**All Authors**: No reported disclosures

